# A Novel, Minimally Invasive Technique in the Management of a Large Cyst Involving the Maxilla in a Child: A Case Report

**DOI:** 10.7759/cureus.2503

**Published:** 2018-04-18

**Authors:** Kishore Moturi, Divya Puvvada, Padma Rayalu Kotha

**Affiliations:** 1 Oral & Maxillofacial Surgery, Vishnu Dental College, Bhimavaram, Ap, India.; 2 Oral & Maxillofacial Surgery, Vishnu Dental College, Bhimavaram, Ap, India; 3 Oral & Maxillofacial Surgery, Dentocare Hospital, Hyderabad, India

**Keywords:** dentigerous cyst, suction catheter, decompression, marsupialization, orthodontic traction

## Abstract

The conservative management of large, odontogenic, developmental cysts involving the jaws in young children using the technique of decompression has become increasingly popular to reduce the incidence of morbidity associated with aggressive treatment modalities. The lack of development of a stock device has made clinicians to explore various possible and readily available materials to customize the devices. This paper highlights the use of a novel material to be used as a decompression device that is readily available and can be used with good patient compliance and a predictable outcome.

## Introduction

The management of cystic lesions of the jaws has evolved over decades, ranging from radical methods, such as enucleation, which involves the removal of the entire cystic lining along with the involved permanent teeth to more conservative approaches, such as marsupialization that involves the creation of a window in the cystic wall to facilitate the spontaneous regression of the cyst or a combination of both, which is described as Partsch procedures. As an extension of this procedure, Thomas, in 1947, introduced the concept of tube drainage, which is popularized as decompression [1].

The treatment of cysts that are odontogenic in origin becomes crucial especially when they originate as developmental lesions affecting the maxilla or mandible in the mixed dentition period. So, the management of these cystic lesions, especially in young children, is of concern with regard to the involvement of permanent teeth buds embedded within the lesion and the adjacent neurovascular bundle. A more conservative means like marsupialization or decompression alone could aid in preserving the vital structures.

We present, in this paper, a case report that was diagnosed as a dentigerous cyst involving the anterior maxilla with multiple, impacted, permanent teeth buds involved in a single follicle and that was managed using a conservative approach in conjunction with orthodontic traction to aid in the eruption of the involved teeth.

## Case presentation

**Figure 1:**
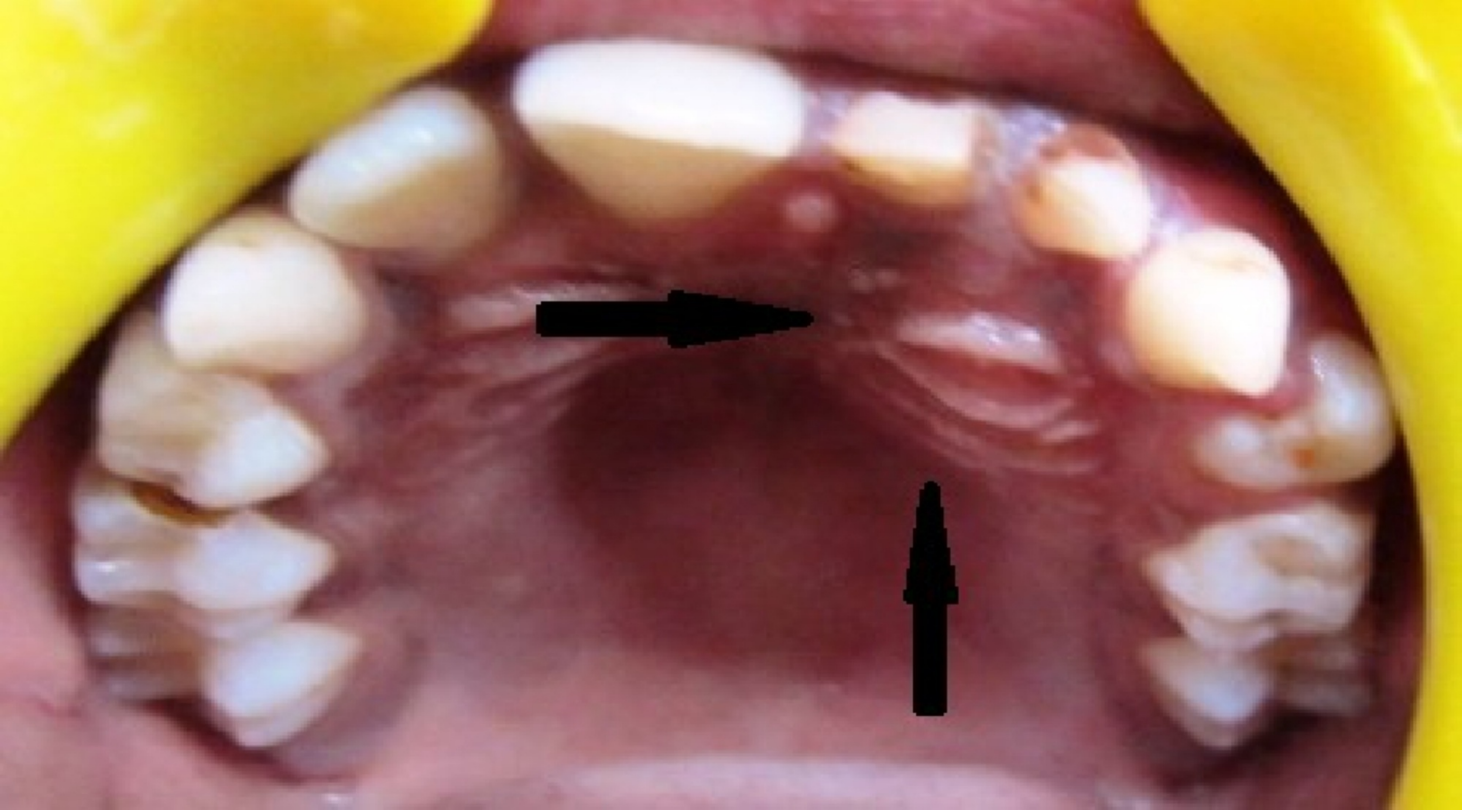
Bicortical expansion of the left anterior maxilla

An 11-year-old boy reported to the department of oral and maxillofacial surgery, Bhimavaram, Andhra Pradesh, India, with a chief complaint of swelling over the left cheek since three months. The swelling was asymptomatic and gradually progressing. A detailed history from the attending parent revealed no significant medical history and no previous history of trauma in the concerned area. On an extraoral examination, there was a gross facial asymmetry on the left side of the face due to the presence of a swelling that extended superiorly from the infraorbital margin to the upper lip inferiorly, obliterating the nasolabial fold. No secondary changes were noticed over the skin. There was no sensory deficit in relation to the facial structures. On a thorough intraoral examination, dentition was mixed, with mild caries affecting the deciduous teeth, none involving the pulp, and revealed a bicortical swelling on the left side extending from the labial frenum medially till the distal aspect of the deciduous second molar, obliterating the labial and buccal vestibule (Figure 1)

**Figure 2:**
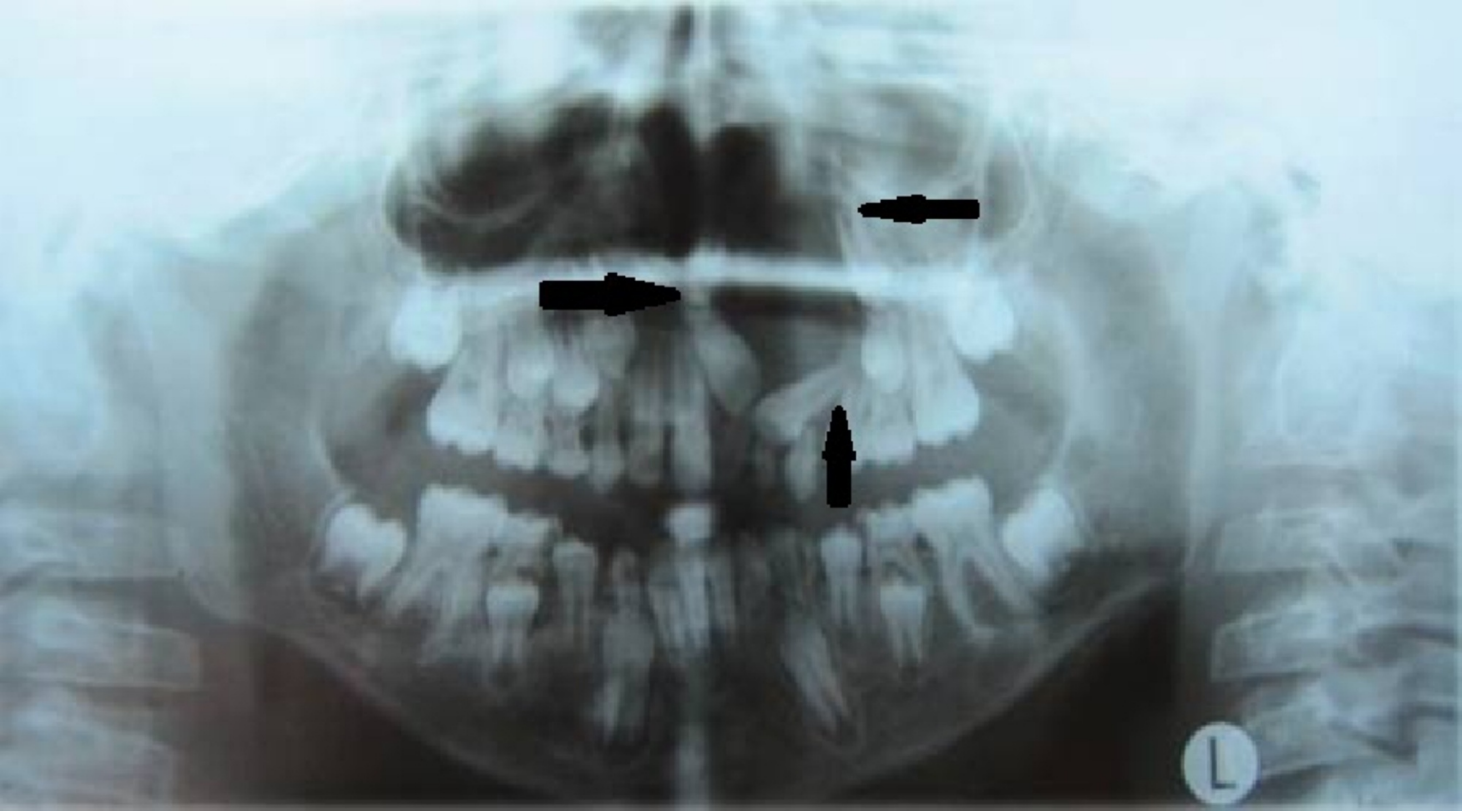
Orthopantomogram shows a cyst involving the left maxilla with an impacted central incisor, lateral incisor, and canine

An orthopantomogram (OPG) revealed a well-defined unilocular radiolucent lesion circumscribing the permanent teeth buds of the central, lateral incisor and canine in the second quadrant (Figure 2). It measured around 3 cm in its greatest dimension.

**Figure 3:**
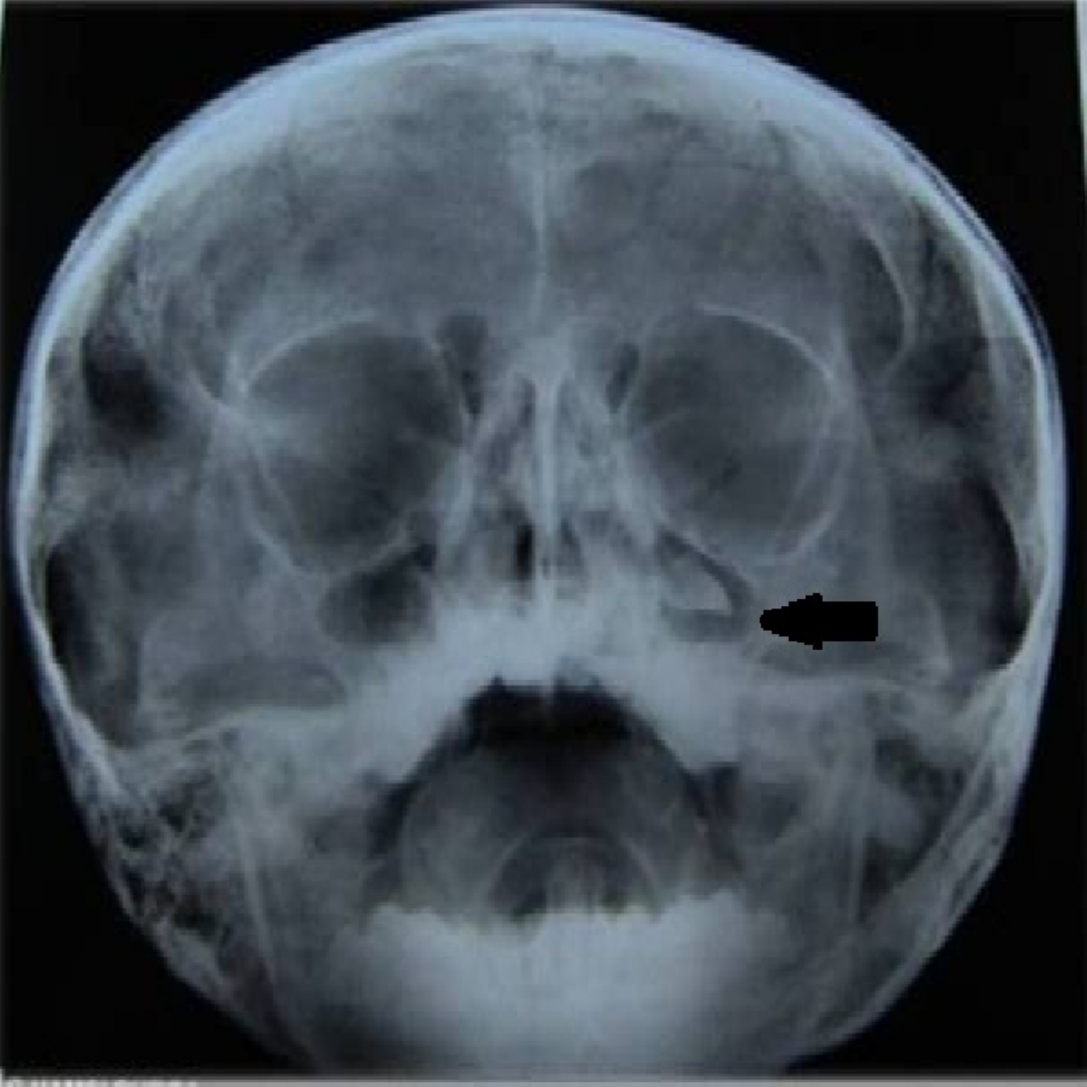
Paranasal sinus view shows the position of the impacted canine near the left infraorbital margin

The paranasal sinus (PNS) view revealed radiolucency extending superiorly till the infraorbital margin with the canine involved (Figure 3). The canine was in Nolla’s stage 7 and the central and lateral incisors were in stage 8.

**Figure 4:**
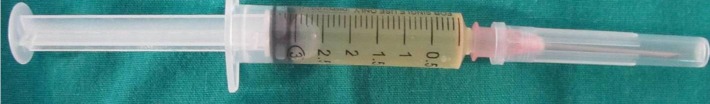
Clear straw-colored fluid obtained on aspiration

Aspiration of the cystic contents revealed a straw-colored fluid. Based on the clinical and radiographic findings, a provisional diagnosis of a dentigerous cyst was made (Figure 4). A differential diagnosis of unicystic ameloblastoma and a cyst involving the maxillary antrum were considered. Based on the above diagnosis, the patient was planned for decompression of the lesion under general anesthesia. After obtaining a procedural and general anesthesia consent from the parents, a thorough pre-anesthetic evaluation was carried out and the obtained routine blood investigation parameters were within normal limits.

**Figure 5:**
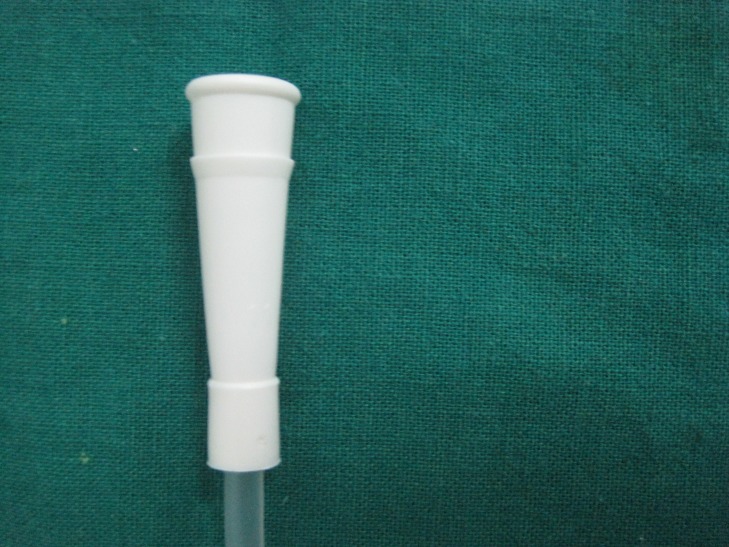
Tip of the suction catheter used as a decompression device

**Figure 6:**
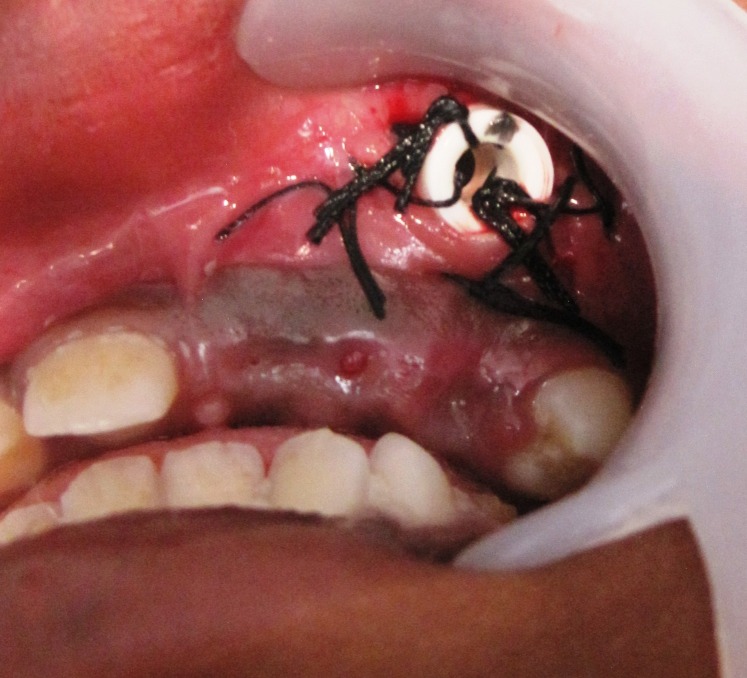
Suction catheter in place, secured with sutures

Under general anesthesia, the extraction of the deciduous central, lateral incisors and canine was done and an opening was created in the anterior wall of the cyst through which part of the lining was removed and sent for histopathological examination. To maintain the patency of the opening, a marsupialization catheter device customized from the tip of a suction catheter (Figure 5) was inserted through the opening and was secured to the margins using 3-0 silk sutures (Figure 6). Parents were instructed to irrigate the cystic cavity using normal saline twice a day for a period of one month, through the opening.

**Figure 7:**
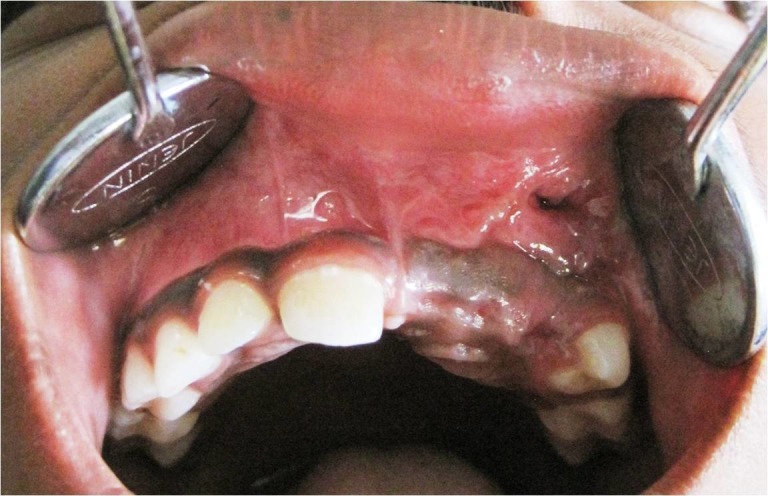
Residual small opening after device removal favoring primary closure

The histopathological report obtained confirmed the diagnosis of a dentigerous cyst. The patient was regularly recalled every week and was assessed for the maintenance of patency through the device. Radiographs were taken at regular intervals to aid in assessing the eruption of the impacted permanent teeth. Device removal was performed after three months (Figure 7).

**Figure 8:**
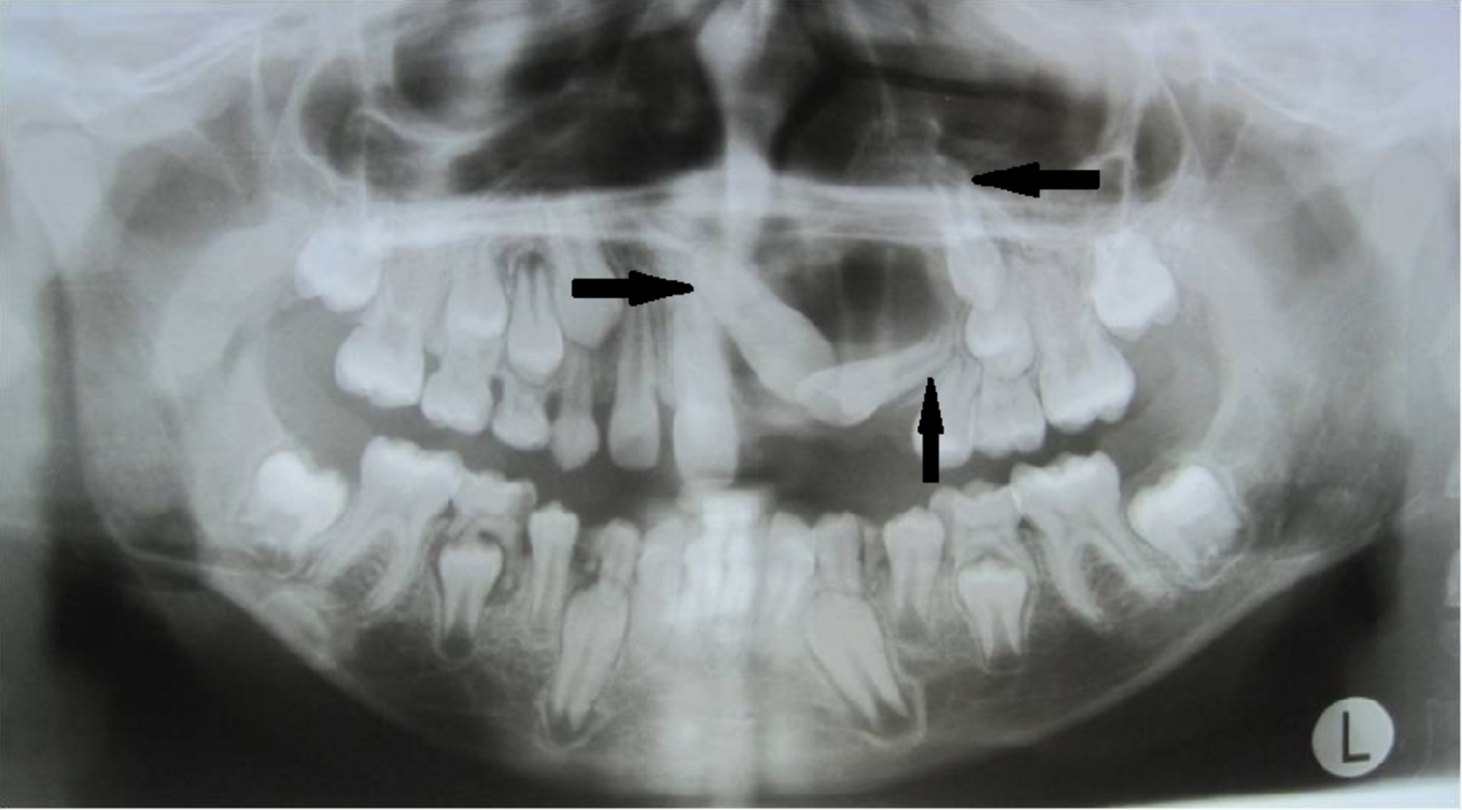
One-month postoperative orthopantomogram (OPG) revealing inferiorly positioned impacted teeth

After a period of three months, the radiographic examination revealed a gradual resolution of the radiolucency of the lesion and a spontaneous eruption of the central and lateral incisors, indicating osteogenesis (Figure 8). So, during a subsequent visit, the patient was referred to the department of orthodontics for the assistive eruption of permanent teeth into the dental arch through traction.

**Figure 9:**
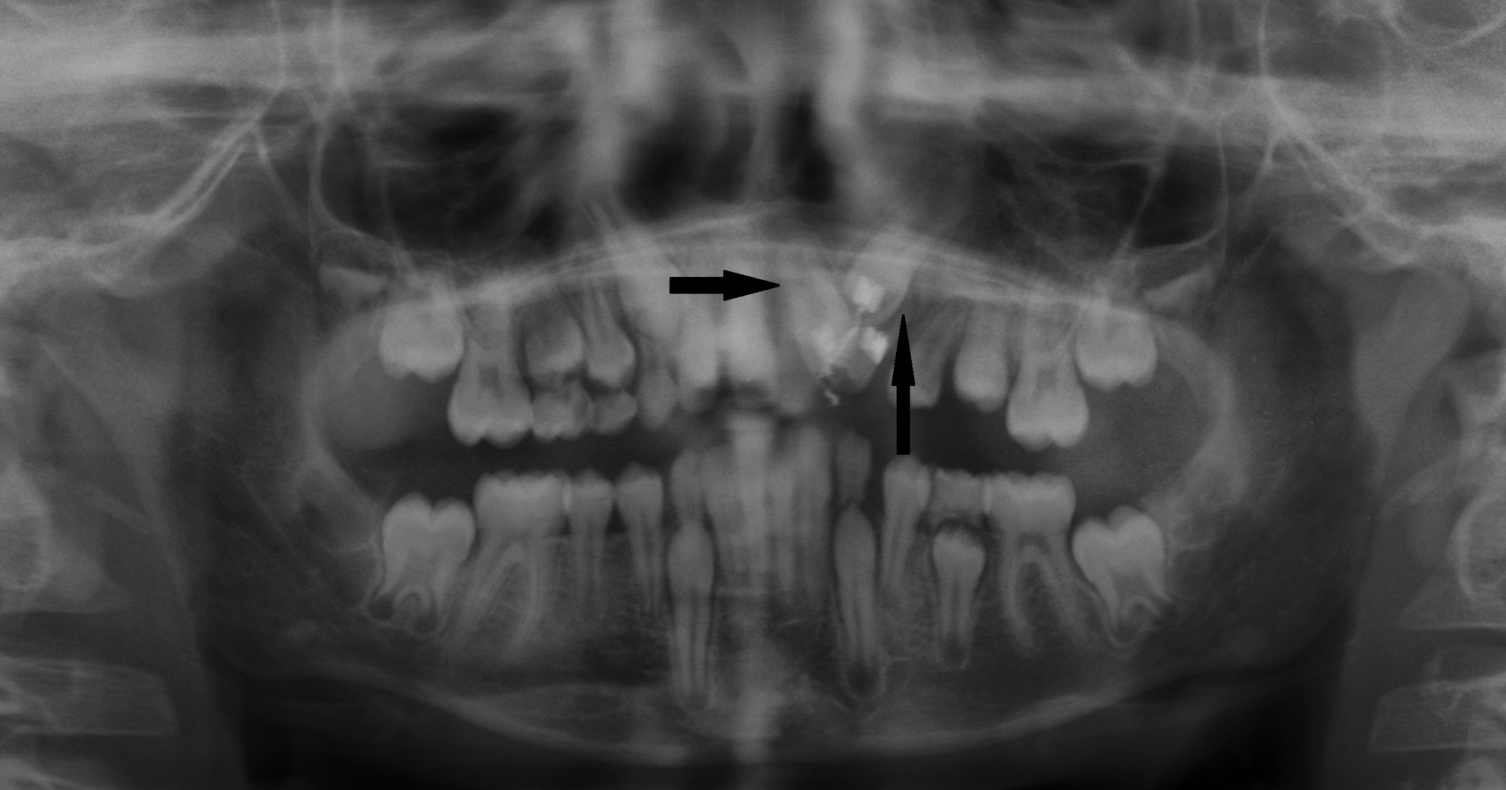
One-year postoperative orthopantomogram (OPG) shows gradual resolution of lesion with erupted central and lateral incisors

**Figure 10:**
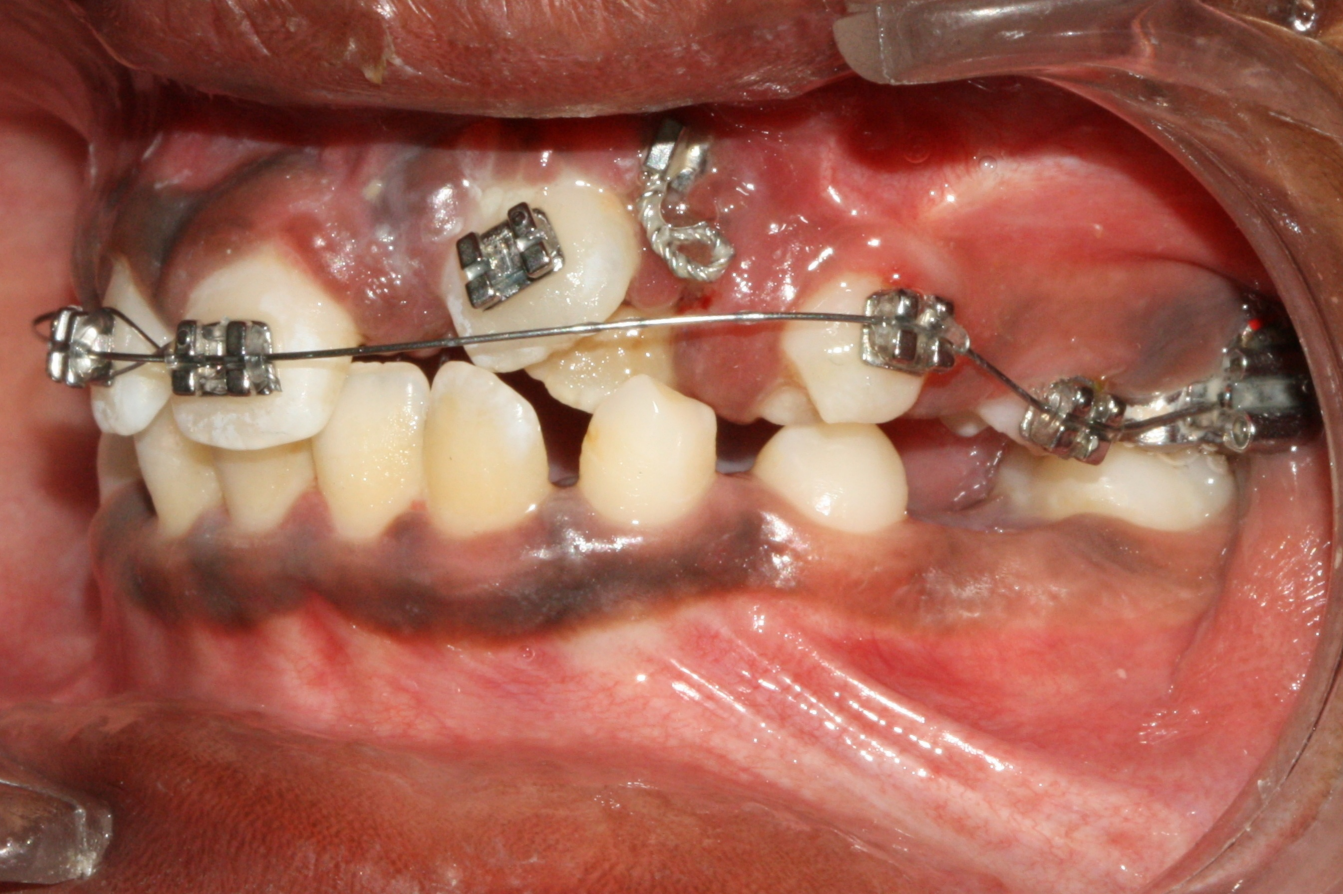
Initial strap up after eruption of central and lateral incisors

**Figure 11:**
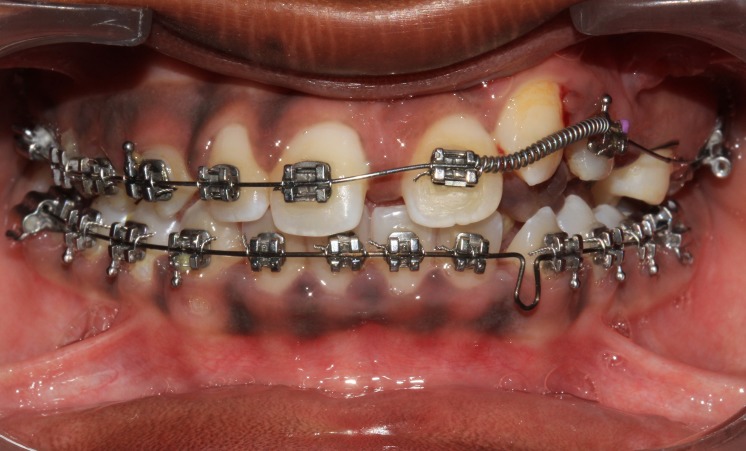
Open coil spring placed to gain space for the canine

**Figure 12:**
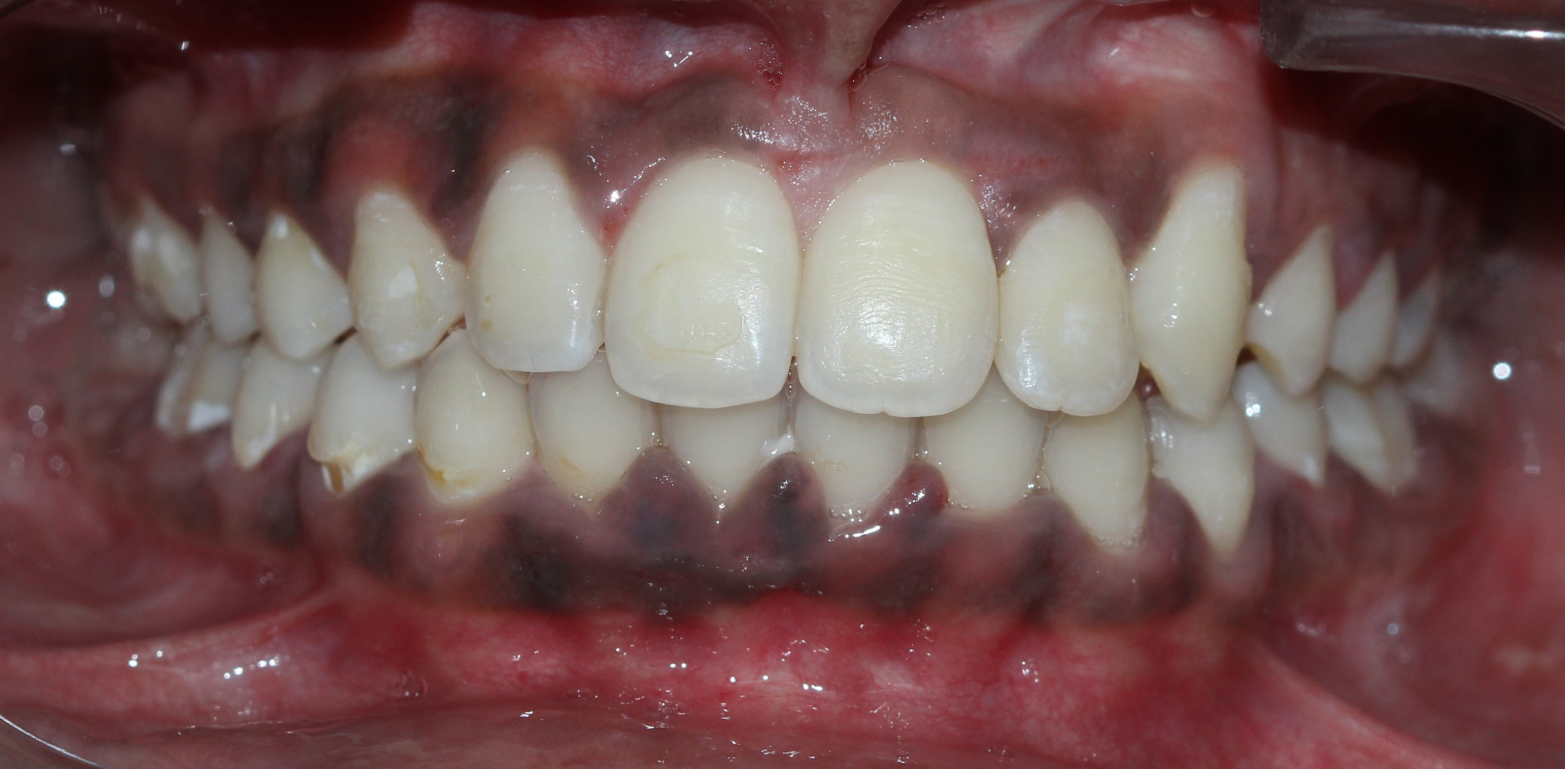
Completely aligned permanent dentition

**Figure 13:**
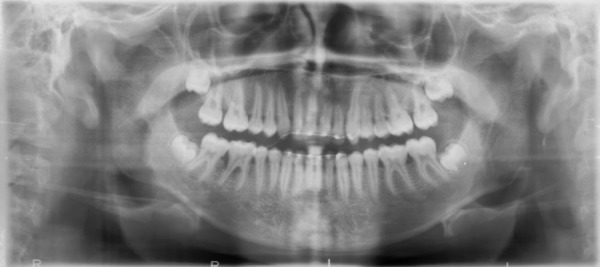
Five years' postoperative orthopantomogram (OPG) showing complete resolution of lesion

Initially, brackets were bonded on the central and lateral incisors and the canine and the flap was closed. Strap-up was done with 0.016 Niti wire. Later, leveling and alignment was done for about six months with 0.0018 Niti 17x25 Niti, 19x25 Niti, 19x25 SS (Figure 9 and Figure 10). After reaching 21x25 SS, wire traction was applied using ligature wire. After the eruption of the central and lateral incisors, an open coil spring was placed for the alignment of the canine after gaining space (Figure 11). The canine was brought into occlusion and finishing and detailing were done. The entire treatment spanned five years for all the three impacted teeth to get aligned in the dental arch (Figure 12 and Figure 13).

## Discussion

**Figure 14:**
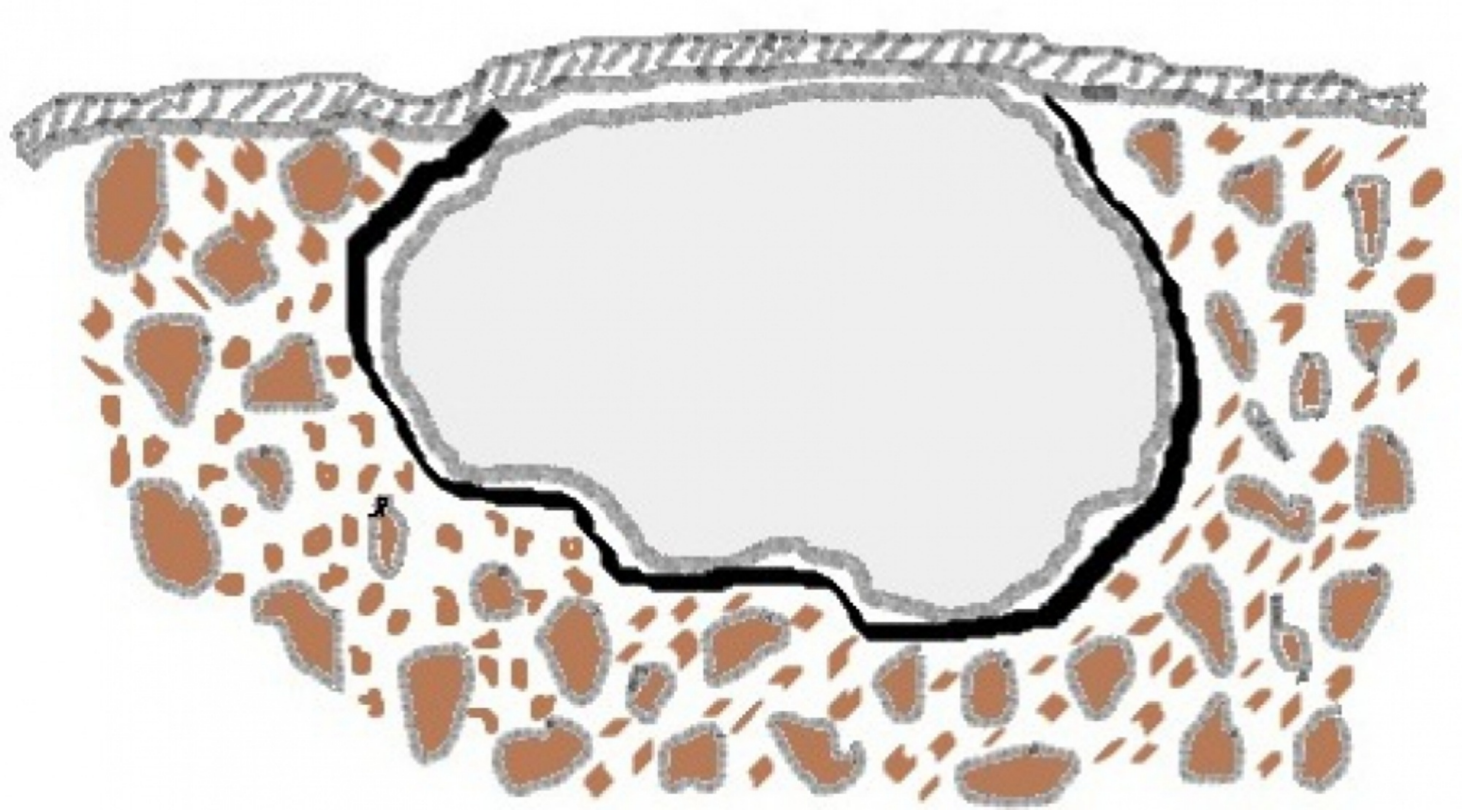
Illustration of cyst within the bone

The decompression of a cyst, by definition, involves any technique that relieves the pressure within the cyst that causes it to grow, as described by Pogrel [2]. It is a phenomenon that occurs in marsupialization where a window is made in the anterior wall of the cyst because of which the osmotic pressure within the cavity is reduced, resulting in a decreased release of bone resorbing factors like prostaglandins, interleukin - alpha, and other growth factors (Figure 14).

**Figure 15:**
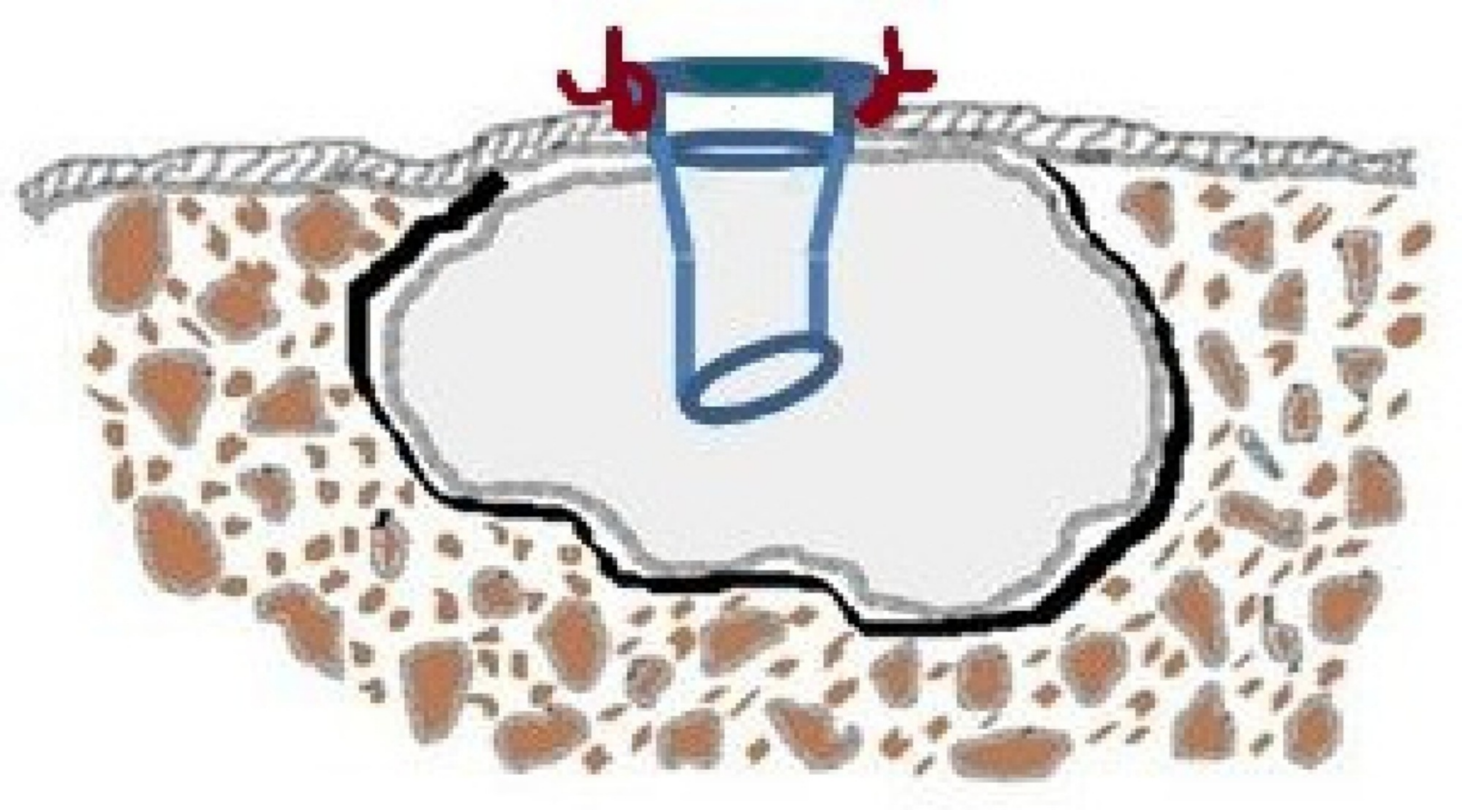
Illustration of decompression tube in place, sutured to the margins of the cystic cavity

**Figure 16:**
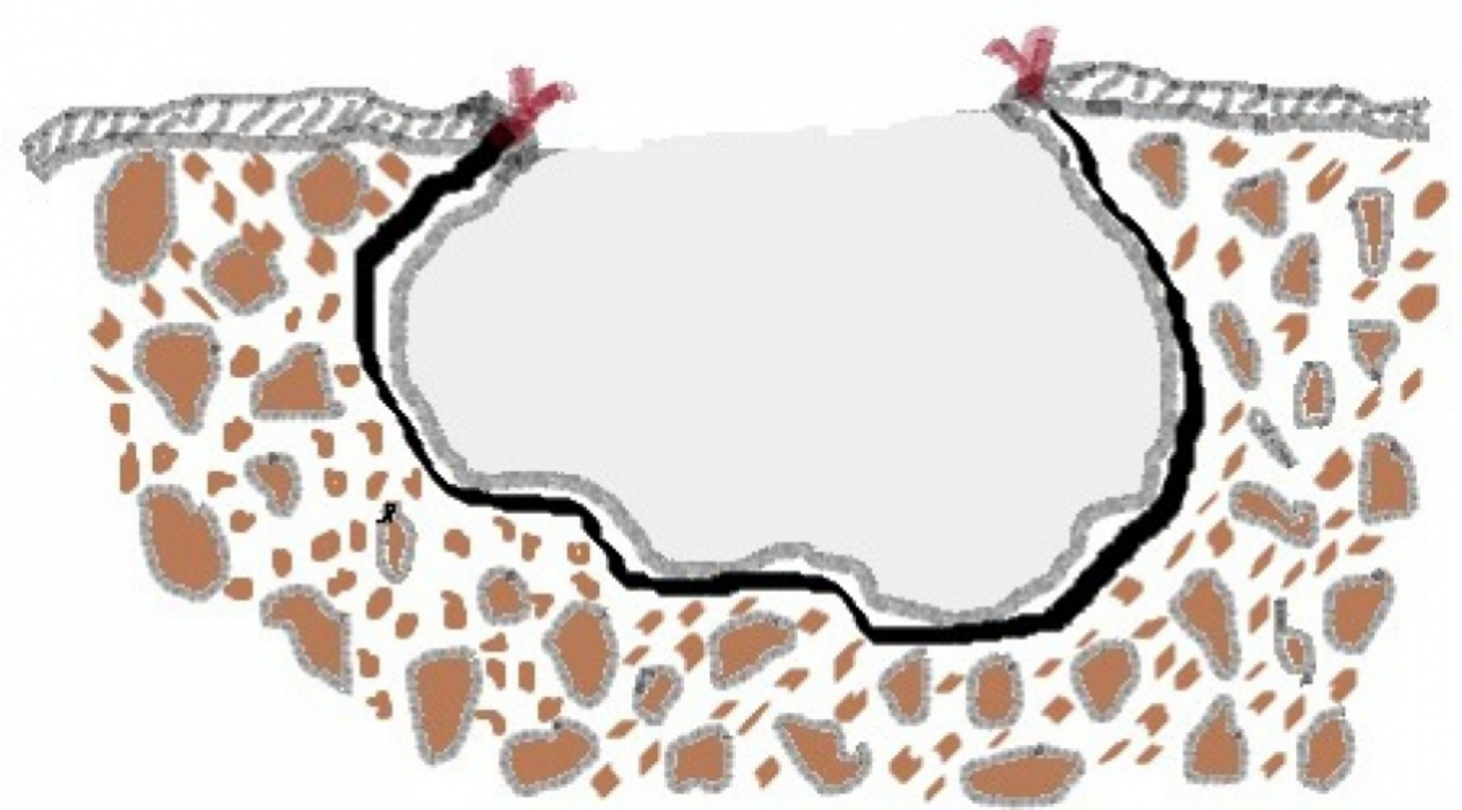
Illustration of the marsupialization procedure

Decompression differs from marsupialization in that the patency of the opening is maintained by placing a tube drain that is secured to margins (Figure 15), while in the latter, a pouch is created by suturing the cystic lining directly to the adjacent normal mucosa without any device (Figure 16). The disadvantage with the marsupialization lies in maintaining the patency of the opening made, which might get covered with the epithelium if the opening made is small. To counteract this, a larger window is made, which can further create trouble obtaining primary closure at a later stage, leaving a residual mucosal defect.

Various catheters have been devised by many authors to serve as decompression tubes that are custom made from intravenous lines, nasal cannulas, pediatric anesthesia tubes, plastic dental syringes, urethral catheters, and a thermoplastic resin to aid in the drainage [1].

Len Tolstunov in 2008 has proposed certain characteristics of devising an ideal decompression tube to be used [3].

1)        Have a design that prevents it from falling into the bone cavity or coming out from it at the end of the procedure;

2)        Be small enough and does not interfere with daily mastication;

3)        Be fixated easily to the soft tissue around it with sutures;

4)        Provide easy daily cleaning of the cystic cavity through its opening by the patient or staff; and

5)        Be hygienic and not accumulate food particles (should not be porous) over the time of its function (weeks and months).

But, none of the custom-made devices satisfies all these ideal characteristics. The lack of development of a stock device has made clinicians explore various possible [4] and readily available materials to customize the devices [5].

The uniqueness of the present case lies in the utilization of a suction catheter as a decompression device. It is a readily available supply that could serve this purpose, although its use has not been reported yet. The advantage of this device lies in its design.

1)    The end of the suction catheter, which is wide at the top and gradually tapers, mimicking a funnel, ensures it does not get easily displaced into the cystic cavity during its usage.

2)    The plasticity of the device enables it to be easily adapted to the opening and be secured with sutures.

3)    It is readily available in the operatory in a sterile pack.

4)    The thickness of the tube prevents the regrowth of tissue over it for a longer period of time.

5)    It is easily acceptable to the patient

All these features make it an exemplary and a feasible device to be used with a good clinical outcome.

The ultimate advantage of using these decompression devices, especially in the younger population to preserve the involved tooth buds, makes it an acceptable technique. The limitation lies in that patient compliance is required during the follow-up extending for a long period of time and proper postoperative care. Hence, this technique is contraindicated in noncompliant individuals.

## Conclusions

The use of a suction catheter proves to be an effective decompression device that can be used with ease and with a predictable outcome without any recurrence of the lesion over a long period of time with good patient compliance. Thus, the authors believe that the device used in this report satisfies all the mentioned requirements of a decompression device for a minimally invasive surgical technique, further favoring its usage.
